# From Policy to Practice: Community Pharmacists’ Self-Reported Counseling Role in Pharmaceutical Waste Management

**DOI:** 10.3390/healthcare14030386

**Published:** 2026-02-03

**Authors:** Ilie Cirstea, Tiberiu Sebastian Nemeth, Delia Mirela Tit, Timea Claudia Ghitea, Ruxandra Cristina Marin, Bogdan Uivaraseanu, Andrei-Flavius Radu, Gabriela S. Bungau

**Affiliations:** 1 Doctoral School of Biological and Biomedical Sciences, University of Oradea, 410087 Oradea, Romania; cirstea.ilie@student.uoradea.ro (I.C.); marin.ruxandracristina@student.uoradea.ro (R.C.M.); andreiflavius.radu@uoradea.ro (A.-F.R.); gbungau@uoradea.ro (G.S.B.); 2 Department of Pharmacy, Faculty of Medicine and Pharmacy, University of Oradea, 410028 Oradea, Romania; timea.ghitea@csud.uoradea.ro; 3 Discipline of Pharmacology, Clinical Pharmacology and Pharmacotherapy, “Carol Davila” University of Medicine and Pharmacy, 050474 Bucharest, Romania; 4 Department of Surgical Disciplines, Faculty of Medicine and Pharmacy, University of Oradea, 410073 Oradea, Romania; buivaraseanu@uoradea.ro; 5 Department of Psycho-Neurosciences and Recovery, Faculty of Medicine and Pharmacy, University of Oradea, 410073 Oradea, Romania

**Keywords:** pharmaceutical waste, community pharmacists, counseling, professional responsibilities, collaborative practice, Romania

## Abstract

**Highlights:**

**What are the main findings?**

- Most community pharmacists report providing disposal counseling, but routine counseling is often reactive.
- Respondents reported high levels of awareness and generally positive attitudes toward pharmaceutical waste prevention.

**What are the implications of the main findings?**

- The frequency of patient inquiries was associated with differences in self-reported counseling practices.
- Community pharmacists continue to have a communication role within the hospital-centered medication take-back system.

**Abstract:**

**Background/Objectives**: Safe disposal of unused medicines represents an increasing public health and environmental concern. Until 2024, Romanian community pharmacies collected expired medicines from the public, though implementation was inconsistent. Using a knowledge–attitude–practice (KAP) framework, this study assessed community pharmacists’ self-reported involvement in pharmaceutical waste prevention in Bihor County, Romania, one year after new legislation transferred collection responsibilities to hospital-based centers. **Methods**: A cross-sectional survey was conducted in May 2025 using a self-administered questionnaire comprising 22 items covering socio-demographics, professional practices, knowledge, and attitudes. Eligible participants were community pharmacists (N = 285). **Results**: Respondents reported high awareness and favourable attitudes toward pharmaceutical waste management: 98.2% indicated awareness of current legislation, 94.4% reported receiving training on the new regulations, 99.6% acknowledged health and environmental risks, and 98.9% expressed agreement that patient education is important. However, 55.4% reported providing disposal information only when patients requested it, while 89.8% indicated that patients rarely asked about medicine disposal. Self-reported proactive counseling increased with patient request frequency (χ^2^(3) = 7.914, *p* = 0.048), with pharmacists in the high-request group reporting substantially higher proactive counseling than those in the low-request group (83.3% vs. 42.9%). In an adjusted logistic regression, low request frequency was associated with lower odds of proactive counseling (aOR = 0.21, 95% CI: 0.05–0.98, *p* = 0.047). Most respondents (94.6%) perceived waste-related responsibilities, though these perceptions were only weakly related to reported counseling behaviors. **Conclusions**: Pharmacists reported high awareness and positive attitudes toward pharmaceutical waste management, but counseling remained reactive. Patient demand was a key correlate of counseling proactivity, underscoring the need for structured education within Romania’s hospital-based take-back system.

## 1. Introduction

Pharmaceutical waste is increasingly recognized as a significant environmental and public health concern. Inappropriate disposal of unused or expired medicines can contaminate water, soil, and food chains, while active pharmaceutical ingredients (APIs) have been detected in river systems worldwide, sometimes at ecotoxicologically relevant concentrations. Several commonly used drug classes, including anti-inflammatory agents, antibiotics, antiepileptics, and psychotropics, are known to persist in wastewater effluents and surface waters due to incomplete removal during conventional treatment, with potential ecological and microbiological implications [1,2]. Such residues have the potential to disrupt microbial ecosystems and disturb ecological balance [3,4].

Managing pharmaceutical waste poses a complex challenge, as medicinal residues persist and circulate through wastewater, surface waters, and other environmental matrices. This persistence underscores the need for effective end-of-life handling policies [5]. Given the diversity of waste sources, both logistical and regulatory complexity are considerable. Institutional pharmaceutical waste is generally handled through regulated systems, while European investigations consistently reveal gaps at the consumer interface, where post-consumer medicines often bypass formal procedures [6,7].

Across health systems, pharmaceutical waste generated in institutional settings is typically classified as healthcare risk waste and managed through regulated streams that emphasize documentation, chain-of-custody control, and approved treatment pathways. Practical guidance highlights standard requirements such as secure storage, labeling, traceability logs, and transport by licensed operators to authorized facilities. High-temperature incineration or equivalent thermal destruction remains the preferred final treatment method, as it minimizes environmental release of active compounds compared with disposal routes designed for general municipal waste [8,9,10].

Household pharmaceutical waste generated through ambulatory therapies requires separate consideration. While institutional pharmaceutical waste in Romania has long been regulated through explicit procedures involving authorized operators, residential pharmaceutical waste historically lacked a cohesive management strategy. In an effort to align with European standards, legislation enacted in 2014 allowed patients to return expired medicines to community pharmacies [11,12].

Earlier Romanian investigations documented the need for systematic solutions at the last stage of product lifecycle management [13] and highlighted household behaviors around disposal [14]. Globally, systematic reviews consistently identify household streams as the weakest component of pharmaceutical waste systems, with high proportions of medicines disposed of via household trash or drains [15,16]. Despite expanded pharmacist responsibilities, the 2014 framework proved difficult to implement, as community pharmacies bore much of the financial and administrative burden, while key elements, collection logistics, transfer mechanisms, and cost-sharing, were insufficiently defined [15,17]. These examples illustrate how system design and convenience features influence participation and the visibility of disposal pathways. Systematic reviews also describe formal medication take-back programs implemented across diverse settings, including pharmacy-based schemes in Europe and Australia and nationally coordinated models in North America [15,17]. In the United States, recurring National Prescription Drug Take-Back Days and permanent drop-off locations improve household access to regulated disposal pathways, while countries such as France and Australia operate nationwide pharmacy-based systems that integrate pharmaceutical waste collection into routine healthcare infrastructure [18]. Across these models, the main objectives include reducing improper household disposal, improving public awareness, and strengthening oversight through regulated return pathways [17].

Romanian case studies from the previous decade documented variable pharmacy engagement and consumer confusion about return options [11,14]. Beyond Romania, recent knowledge, attitude and practice (KAP) surveys continue to identify inconvenience and limited access to take-back points as major barriers to correct household disposal [19,20]. Cross-sectional studies indicate that although knowledge and attitudes toward safe disposal are generally favorable, actual disposal practices remain suboptimal l [21]. Systematic reviews of KAP-based research similarly report persistent gaps between attitudes and practices among both the general public and healthcare professionals, underscoring the value of KAP approaches for identifying modifiable barriers and informing educational and infrastructural interventions [7].

Community pharmacists occupy a central position between public expectations and structural constraints. As accessible healthcare professionals, they are often approached by patients seeking advice on expired medicines, yet their ability to manage returns and provide systematic counseling has historically been limited by the absence of standardized tools and institutional support. Recent studies reinforce pharmacists’ important but often under-resourced role in pharmaceutical waste prevention and patient education [22,23,24], consistent with life-cycle analyses showing that the end-of-life stage remains the least integrated component of product stewardship [13].

In 2023–2024, Romania adopted a new policy framework that fundamentally restructured pharmaceutical waste management. Under Law No. 269/2023 (amending Law No. 95/2006) [25] and subsequent Instruction No. 6226/2024 [26], responsibility for collecting expired or unused medicines from the population was transferred to public and private hospitals, which are legally obliged to receive them for final elimination. The legislation also introduced mandatory on-pack warnings directing patients to return unused medicines to hospitals [27], while budgetary and reimbursement mechanisms were clarified through subsequent regulatory orders [28]. Together, these measures centralized collection and clarified both operational and financing responsibilities.

From a public health perspective, centralization may enhance regulatory control and traceability by consolidating disposal pathways within audited systems and reducing fragmentation. Systematic reviews of take-back programs indicate that such centralized mechanisms improve transparency and accountability compared with unregulated household disposal [8,17]. Nevertheless, international evidence shows that accessibility and convenience remain critical determinants of household participation, and improper disposal practices often persist when return points are perceived as distant or inconvenient [16,17]. Within this evolving context, the preventive and educational role of community pharmacists remains essential. Although hospital-based collection relieves pharmacies of direct take-back logistics, the pharmacist–patient interface remains central for prevention, counseling, and directing patients toward appropriate return pathways. Studies show that pharmacist-led communication improves awareness but is often constrained by limited training, lack of materials, and uncertainty regarding responsibilities [22,23]. Given the continued prevalence of household disposal through regular waste channels [15], understanding how pharmacists interpret and enact their role under the revised framework is critical.

Behavioral frameworks for healthcare professionals emphasize that the implementation of preventive practices depends on more than knowledge and attitudes, reflecting a broader behavior system shaped by capability, opportunity, and motivation [29]. In pharmacy settings, qualitative evidence from medication take-back initiatives highlights practical constraints and enabling conditions, such as staffing levels, financial and geographic barriers, facility availability, and knowledge limitations, alongside facilitators including professional responsibility, incentives, and convenient locations [22]. Within this framework, patient-initiated questions represent an opportunity for counseling and may help explain differences in how consistently disposal guidance is provided in routine practice.

Against this background, the present study surveyed community pharmacists in Bihor County, Romania, approximately one year after the implementation of the hospital-centered take-back system, to assess knowledge, attitudes, and reported counseling practices regarding pharmaceutical waste prevention. The study examined whether pharmacists who receive patient questions more frequently also report more proactive disposal counseling, while adjusting for practice context and role-related factors. Although locally focused, the study provides context-specific evidence to inform implementation and public-facing communication under the revised national framework.

## 2. Materials and Methods

### 2.1. Study Design

This was a cross-sectional study conducted to evaluate the perceptions and practices of Romanian community pharmacists in relation to pharmaceutical waste prevention.

Data collection took place in May 2025, more than one year after the implementation of updated national legislation (Law 269/2023), which shifted the responsibility for expired medicine collection from community pharmacies to hospital-based centers. This timeline was chosen to ensure that pharmacists’ responses reflected current roles and responsibilities under the new framework.

A self-administered questionnaire was used in printed format. Although an online version was initially piloted, all finalized data were collected using paper questionnaires distributed on-site. Before completion, all participants received an introductory section describing the study’s purpose, voluntary participation, confidentiality, and the intended use of results. Informed consent was recorded at the beginning of the questionnaire; respondents indicated agreement by providing the date and a short confirmation on the form. No names, contact details, or professional registration identifiers were collected. Responses were analyzed in aggregate; however, as with most surveys, combinations of demographic variables could theoretically allow indirect identification.

The analytical approach was grounded in a behavioral framework, recognizing that pharmacists’ preventive practices may reflect interactions between knowledge, attitudes, and contextual opportunity. The KAP structure guided the assessment of regulatory awareness, attitudes toward pharmaceutical waste prevention, and self-reported counseling behaviors. This framework also informed expectations about how these dimensions might interact under the reorganized take-back system introduced by the 2023–2024 legislation.

Within this conceptual model, it was expected that pharmacists with greater awareness of current legislation and those who perceived waste-related activities as part of their professional responsibilities would be more likely to report counseling behaviors. It was further anticipated that positive attitudes toward patient education would align with support for proactive, in-pharmacy informational measures. Given the central role of the pharmacist–patient interface, counseling activity was also expected to correlate with being approached by patients regarding unused or expired medicines. This conceptual foundation guided variable selection and interpretation of relationships among study indicators.

### 2.2. Population and Sample

This study targeted community pharmacists practicing in Bihor County, Romania (N = 803, officially registered according to the county pharmacist registry). Initially conceptualized as a nationwide online survey, the project encountered feasibility constraints due to a very low response rate (<5%) obtained through online pharmacist forums and professional networks. Consequently, the recruitment strategy was adapted to a venue-based convenience sampling design focused on a single administrative unit.

The revised approach was implemented during a major regional professional pharmacy event held in May 2025. The event was organized by the county-level professional college and was open exclusively to licensed pharmacists and final-year pharmacy students, the latter granted free entry. Printed questionnaires were distributed on-site, and participation was voluntary with confidential handling of responses.

The target population for this analysis comprised community pharmacists practicing in Bihor County. Inclusion criteria were self-reported pharmacist qualification and current practice in Bihor County. Exclusion criteria included pharmacy assistants, respondents practicing outside the target county, and questionnaires lacking essential data for the main study variables. Some final-year students may have held pharmacy assistant qualifications, which could explain the small number of assistant-completed questionnaires that were excluded.

**Figure 1:**
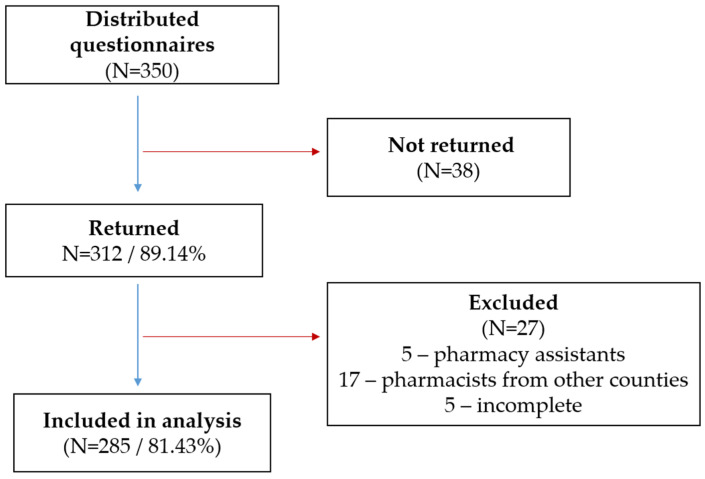
Flow diagram of participant recruitment, response rate, and inclusion in final analysis.

Of the 350 questionnaires distributed, 312 were returned (response rate 89.14%), and 285 were included in the final analysis (81.43%). Based on self-reported role and qualifications, 27 questionnaires were excluded: 5 submitted by pharmacy assistants, 17 by pharmacists practicing in other counties, and 5 due to incomplete data. A visual flow diagram of this process is provided in Figure 1.

No probabilistic or random sampling was employed; thus, no design effect correction or precision-based sample size calculation is applicable. While the number of responses exceed typical thresholds for descriptive studies, the sample should not be interpreted as statistically representative of the entire pharmacist population in Bihor County or Romania. Selection bias may be present, as participation was limited to those attending the event and willing to complete the questionnaire. Social desirability bias may also influence self-reported practices regarding pharmaceutical waste.

### 2.3. Data Collection Instruments

The questionnaire was developed by a multidisciplinary team of pharmacists and public health researchers, grounded in a comprehensive review of international literature on pharmaceutical waste management, pharmacist-led counseling interventions, and sustainability practices [23,30,31,32,33]. It was also informed by recent changes in the Romanian regulatory framework, including Law 269/2023 [25] and Instruction 6226/2024 [26].

The draft questionnaire underwent expert review by two pharmacy academics and one public health policy advisor to ensure content validity and assess clarity, relevance, and conceptual scope. A pilot test was subsequently conducted with 20 pharmacists from outside Bihor County to evaluate item comprehension and usability. Only minor wording adjustments were required, and no structural modifications were made. These steps aimed to minimize measurement bias by improving clarity and reducing misinterpretation. The questionnaire introduction emphasized voluntary participation and confidentiality to limit social desirability bias.

The final instrument contained 22 items organized into three thematic sections: items 1–9 captured socio-demographic and professional characteristics; items 10–14 addressed practice- and role-related aspects; and items 15–22 assessed knowledge and attitudes relevant to pharmaceutical waste prevention, following the KAP structure. Professional categories reflect the Romanian framework: pharmacy assistant (technical role), pharmacist (licensed university-trained professional), and specialist/primary pharmacist (advanced professional grades based on postgraduate training and/or seniority). Table 1 summarizes the analyzed items and their response formats, while the full questionnaire wording and administration order are provided in Supplementary Material S1.

### 2.4. Data Analysis

Statistical analyses were conducted using JASP software (version 0.19.3). Categorical variables were summarized as frequencies and percentages, and continuous variables as mean ± SD.

Given the high prevalence of affirmative responses to the binary counseling item, counseling behavior was operationalized primarily using the frequency-based measure. Counseling was dichotomized as reactive (information provided only upon request) versus proactive (information provided rarely, almost always, or always). The frequency with which patients asked for disposal guidance was analyzed both as an ordered predictor (never to always) and as a dichotomous exposure for interpretability (low: never/rarely; high: almost always/always).

Bivariate associations were examined using contingency tables with Pearson’s chi-square tests; Fisher’s exact test was additionally applied for 2 × 2 comparisons when expected counts were small. For 2 × 2 contrasts, prevalence ratios (PR) and risk differences (RD) were computed to aid interpretation alongside odds ratios (OR). Ordinal trends were evaluated using logistic regression with patient-request frequency entered as an ordered predictor.

To assess whether the association between patient requests and counseling proactivity persisted after accounting for practice context, a multivariable logistic regression model was fitted with robust (sandwich) standard errors. The adjusted model included the dichotomized patient-request indicator (high vs. low) and a small set of a priori covariates: practice setting, pharmacy type, and years of experience. Adjusted odds ratios (aOR) with 95% confidence intervals (CI) were reported, and model-based predicted probabilities were used for graphical presentation. Analyses were conducted on complete-case data for each model.

Associations between declared waste-related responsibilities and binary indicators of counseling and patient approaches were assessed using chi-square tests, with effect sizes reported as Cramer’s V. Exploratory interrelations among selected knowledge, attitude, and practice indicators were examined using Spearman’s rank correlation coefficient (ρ).

All tests were two-sided, with a significance level of *p* < 0.05 used as a reference threshold. Analyses were exploratory, and no adjustment for multiple comparisons was applied.

## 3. Results

### 3.1. Socio-Demographic and Professional Characteristics of Respondents

The sample (N = 285) reflected a predominantly experienced pharmacist cohort, with a mean age of 41.2 ± 9.4 years and 81.8% reporting more than ten years of professional experience. The gender distribution was strongly skewed toward females (91.2%), consistent with the feminization trend in the Romanian pharmacy workforce. Most respondents practiced in urban settings (69.5%), while 30.5% worked in rural areas. Formal educational attainment was dominated by a bachelor’s degree in pharmacy (82.5%), followed by postgraduate qualifications: master’s degrees (8.1%), residency training (5.6%), and doctoral studies (3.9%). Professional grade distribution mirrored this profile, with most respondents holding the basic pharmacist qualification (92.6%), while smaller proportions held advanced professional grades such as specialist (3.5%) or primary pharmacist (3.9%). Pharmacy ownership and affiliation showed considerable diversity: 42.5% of respondents worked in local pharmacy chains, 31.2% in national chains, and 26.3% in independent pharmacies. Regarding job position, 81.8% were staff pharmacists and 18.2% were head pharmacists (Table 2).

### 3.2. Knowledge, Attitudes and Counseling Practices Regarding Pharmaceutical-Waste Management

#### 3.2.1. Knowledge and Attitudes

Almost all respondents reported familiarity with the current national legislation on pharmaceutical waste disposal (98.2%) and professional training on the relevant regulations (94.4%). Awareness of the potential health and environmental risks associated with improper disposal was nearly universal (99.6%), and 95.4% believed that incorrect disposal of antibiotics contributes to antimicrobial resistance. A very high proportion of pharmacists considered patient education on correct disposal important (98.9%) and viewed proactive in-pharmacy promotion as useful (97.9%).

Despite these favorable attitudes, reported counseling behaviors were largely reactive. More than half of respondents stated that they provide disposal-related information only when patients request it (55.4%), while most reported that patients rarely ask about this topic (89.8%). This distribution suggests that, although awareness and attitudes toward safe disposal are strong, counseling on pharmaceutical waste remains insufficiently integrated into routine pharmacy practice (Table 3).

#### 3.2.2. Counseling Proactivity and Patient Demand

To capture variation in counseling practices beyond the near-universal endorsement of counseling provision, counseling behavior was operationalized using the frequency-based item (Q17). Reactive counseling was defined as providing information only upon patient request, whereas proactive counseling was defined as providing information rarely, almost always, or always. Patient request frequency (Q18) was analyzed both as a four-level ordinal variable and as a dichotomized indicator for interpretability.

A graded association was observed between patient request frequency and counseling proactivity (Table 4). Patient request frequency differed significantly between proactive and reactive practitioners, with higher levels of patient demand being more common among those engaging in proactive counseling (χ^2^(3) = 7.91, *p* = 0.048). In a logistic regression treating Q18 as an ordered predictor (coded 1–4), higher patient request frequency was associated with greater odds of proactive counseling (OR = 2.19, 95% CI [1.04–4.60]; *p* = 0.038).

For the dichotomized comparison (low: never/rarely vs. high: almost always/always), proactive counseling was reported by 83.3% (10/12) of pharmacists in the high-request group compared with 42.9% (117/273) in the low-request group (χ^2^(1) = 7.623, *p* = 0.006; Fisher’s exact *p* = 0.007) (Table 5). The unadjusted prevalence ratio for proactive counseling (high vs. low) was 1.94 (95% CI 1.46–2.59), and the absolute difference was +40.5 percentage points (95% CI +18.6 to +62.4).

**Figure 2:**
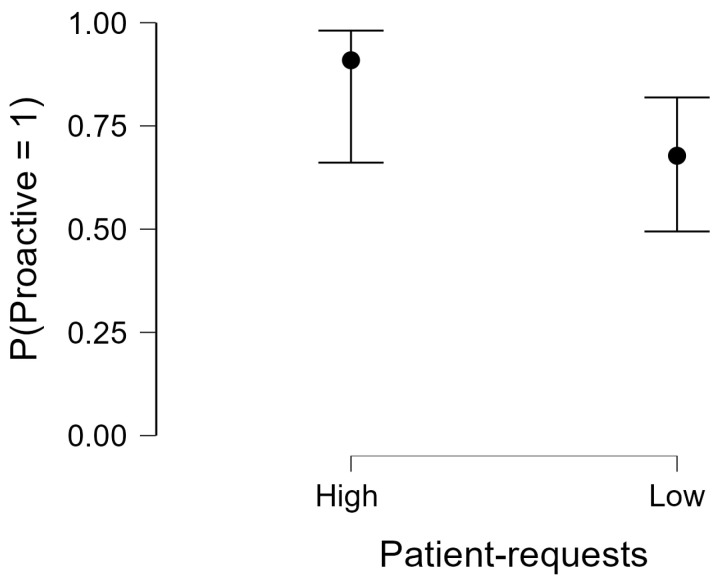
Adjusted predicted probability of proactive counseling by patient request frequency (high vs. low). Points represent model-based predicted probabilities from a multivariable logistic regression with robust standard errors; error bars indicate 95% confidence intervals. The model adjusted for setting, pharmacy type (chain vs. independent), and experience (>10 vs. ≤10 years).

In the adjusted logistic regression model with robust standard errors, lower patient-request frequency remained independently associated with lower odds of proactive counseling compared with high request frequency (aOR = 0.21, 95% CI [0.05–0.98]; *p* = 0.047). Model-based predicted probabilities are presented in Figure 2.

### 3.3. Counseling Behavior, Patient Requests and Perceived Responsibilities

In the total sample (N = 285), 280 pharmacists (98.2%) indicated that they counsel patients on the safe disposal of expired or unused medicines, while only 5 (1.8%) reported not providing such counseling. Among those who counselled, most also reported being approached by patients with disposal-related questions (268/280, 95.7%), compared with 2/5 (40.0%) among the non-counseling group. This difference should be interpreted with caution due to the very small number of non-counseling respondents.

Perceived involvement in pharmaceutical waste reduction was also high. Among respondents with complete data (N = 280), 265 (94.6%) reported having explicit waste-related responsibilities. Associations between declared responsibilities and the binary variables representing counseling provision and patient approach were weak (Table 6), with small between-group differences and low effect sizes (Cramer’s V = 0.105, *p* = 0.079 for counseling; Cramer’s V = 0.023, *p* = 0.702 for patient approach). Because of the limited discriminatory power resulting from highly uniform responses, these statistical associations should be interpreted cautiously and viewed as exploratory.

### 3.4. Interrelations Between Knowledge, Attitudes and Counseling Practice

To assess the relationships among key knowledge, attitude, and self-reported practice indicators, Spearman’s rank correlation coefficients (ρ) were calculated.

A higher level of training on the new regulations tended to be associated with self-reported awareness of current pharmaceutical waste disposal legislation (ρ = 0.548, *p* < 0.001). A positive association was also observed between considering patient information important and viewing proactive in-pharmacy promotion as useful (ρ = 0.706, *p* < 0.001), reflecting internal consistency among attitude-related items.

Perceived awareness that incorrect disposal of antibiotics contributes to antimicrobial resistance showed moderate positive correlations with both the importance attributed to informing patients (ρ = 0.321, *p* < 0.001) and the perceived usefulness of proactive promotion (ρ = 0.227, *p* < 0.001). In contrast, the correlation between the frequency of providing disposal information and the importance assigned to informing patients was weak and negative (ρ = −0.143, *p* = 0.016), suggesting that favourable attitudes do not necessarily translate into more frequent counseling. These correlation analyses were exploratory and interpreted descriptively given the limited variability and high endorsement rates across several dichotomous items. (Table 7).

## 4. Discussion

In Romania, the regulatory framework for the disposal of unused medicines has undergone a major transition. Until recently, community pharmacies were responsible for collecting expired medicines returned by the population under Order No. 119/2014 of the Ministry of Health [34]. Following the adoption of Law No. 269/2023 and Instruction No. 6226/2024, this responsibility was transferred to specialized collection centers organized within public and private hospitals. This reform addressed long-standing concerns regarding unclear responsibilities and the financial and administrative burden placed on pharmacies [25,26]. Such clarification of ownership aligns with international evidence indicating that take-back systems function most effectively when responsibilities and financing mechanisms are explicitly defined and operationalized [17,35].

In this evolving context, the present study offers insight into how Romanian community pharmacists perceive and report their involvement in pharmaceutical waste prevention during the early implementation phase of the new policy. Although pharmacies were no longer legally mandated to collect medicines at the time of data collection (May 2025), most pharmacists continued to counsel patients on disposal and to be approached for guidance. This pattern suggests a persisting public expectation that pharmacies remain a primary source of advice, reflecting a transitional professional role that has not yet fully adapted to the revised legal framework.

From a knowledge–attitudes–practice (KAP) perspective, pharmacists in Bihor County reported high levels of knowledge and favorable attitudes toward pharmaceutical waste management. Most respondents indicated awareness of the current legislation, reported having received training on the new regulations, acknowledged environmental and health risks, expressed support for patient education and proactive promotion. Because most knowledge and attitude items showed ceiling effects, their discriminatory value was limited. These results are consistent with national and international surveys showing high pharmacist awareness of pharmaceutical pollution and antimicrobial resistance [22,23,24,36,37]. However, as noted in prior systematic reviews, high awareness does not necessarily translate into consistent practice when infrastructure and workflow integration are lacking [7,38].

In this study, more than half of respondents provided disposal information only when patients requested it, while most reported that such requests were rare, indicating that counseling remains largely reactive rather than routinely integrated into daily practice. Correlation analyses further supported this interpretation. Training on the new regulations was strongly associated with self-reported awareness of current pharmaceutical waste legislation, and attitudes regarding patient information and proactive in-pharmacy promotion were closely aligned. Similar findings have been documented in other KAP studies, where pharmacists acknowledging environmental risks tend to support structured patient-information initiatives [24,39,40]. Together, these results indicate that favorable knowledge and attitudes are widespread, but proactive counseling is most likely to occur when pharmacists encounter patient demand, suggesting that patient-initiated questions act as a behavioral trigger in daily practice.

The observed associations between perceived responsibilities, counseling activity, and patient requests appear to support this interpretation. However, these relationships should be interpreted cautiously, given the limited variability and small group differences observed across variables. Although nearly all pharmacists reported formal responsibility for pharmaceutical waste reduction, these perceptions were weakly related to actual counseling or patient interactions. This suggests that everyday pharmacist–patient exchanges are influenced more by visibility and patient demand than by formal definitions of professional roles. This interpretation aligns with comparative evidence showing that accessibility and ownership clarity are critical determinants of system performance [17,41,42].

The analyses focusing on counseling proactivity and patient request frequency provide the study’s most informative empirical finding. Pharmacists who reported more frequent patient requests were significantly more likely to engage in proactive counseling on disposal, with a clear graded pattern across response categories. This association remained evident in both dichotomized and adjusted models, although the high-request subgroup was small and estimates should be interpreted with caution. These results are consistent with established KAP frameworks in which knowledge and attitudes interact with perceived demand and contextual opportunities to shape counseling behavior [7,24,36,37]. However, given the limited variability and the predominance of highly endorsed items, these relationships should be regarded as exploratory patterns rather than explanatory mechanisms. In this setting, knowledge and attitudes were almost uniformly favorable, yet proactive counseling was more common among pharmacists who encountered higher patient demand. Rather than indicating a lack of awareness or willingness to counsel, the findings suggest that routine opportunities created by patient questions may serve as key behavioral cues in everyday practice.

Taken together, these findings point to a visibility–demand linkage, whereby pharmacist engagement and patient demand reinforce each other. Evidence from intervention syntheses indicates that pharmacist-delivered prompts, such as brief advice, printed instructions, or reminders, can improve disposal behavior, particularly when paired with clear and accessible return pathways [41,42,43]. In the current post-policy context, where pharmacies no longer collect expired medicines but remain highly accessible information points, such communication functions may be particularly important for connecting patients with hospital-based take-back options. Nonetheless, Spearman correlations also highlighted a modest knowledge–practice gap, consistent with other KAP studies in which time constraints, competing priorities, lack of standardized workflows, and low patient demand limit systematic counseling despite strong professional commitment [24,36,37,39,40]. Broader evidence suggests that workflow constraints and limited access to clearly signposted collection infrastructure perpetuate patterns of favorable attitudes combined with inconsistent practice across healthcare professionals [7,41,42].

These findings indicate that interventions aimed at improving pharmaceutical waste management should focus not only on increasing knowledge but also on restructuring the conditions under which counseling occurs. Low-burden, workflow-integrated tools—such as standardized counseling phrases, prompts within dispensing software, or ready-to-use information leaflets—may help shift counseling from a reactive to a more proactive component of routine care, particularly when combined with clear return pathways and repeated exposure [17,42]. In parallel, prevention-oriented strategies, including pack-size optimization, stop-rules for short courses and adherence checks, remain high-yield approaches for reducing unused medicines at source [44].

From an environmental and public health perspective, residues from medicines and nutraceuticals may persist in soil and aquatic systems and contribute to ecotoxic effects and antimicrobial resistance [3,4,5,45,46]. The combination of avoidable oversupply, stockpiling, and improper disposal reinforces the importance of pharmacist-led interventions not only at the end-of-life stage but also upstream, at prescribing, dispensing, and self-care decision points. Current literature highlights that upstream interventions primarily target the prevention of unnecessary medicine acquisition and accumulation at the household level. These include optimizing prescription quantities and durations, improving communication on intended therapy length, and avoiding automatic refills without clinical reassessment. Reviews consistently identify inappropriate prescribing, therapy changes, non-adherence, and premature discontinuation as major drivers of unused medicines, underscoring the pharmacist’s role in supporting adherence and rational use before surplus occurs [1,15]. Recent work conceptualizing non-adherence as a key driver of pharmaceutical waste further emphasizes the relevance of adherence-focused interventions, such as patient education, regimen simplification, medication optimization, and deprescribing when appropriate, which can reduce both the volume of unused medicines and the downstream burden on take-back and disposal systems [47]. By integrating adherence checks, pack-size optimization, explicit stop-rules for short-term therapies and clear advice on what to do with leftover medicines, pharmacists can help reduce waste generation upstream, while counseling on safe disposal addresses downstream risks [48,49,50]. This dual framing of prevention and safe return is echoed in recent policy and review literature [17,35,44].

The findings have important implications for the implementation of Romania’s hospital-centered take-back system. International evidence shows that effective take-back programs combine accessible return routes, clearly allocated ownership and financing, and behaviorally informed communication strategies [17]. Beyond behavioral determinants, system performance is heavily influenced by governance and operational factors across the reverse-logistics chain. Sustainable systems rely on defined accountability frameworks, adequate infrastructure, reliable collection and transport logistics, and sufficient downstream treatment capacity. Additionally, traceability information systems, stable financing mechanisms, and inter-institutional coordination have been identified as essential for continuity and regulatory oversight [51,52,53].

European experience reinforces these principles. Established programs in Sweden, France, and Spain demonstrate that sustained public information campaigns and structured return networks can achieve substantial collection volumes and recovery rates [54,55,56]. EU legal analyses further highlight the importance of harmonized and clearly assigned responsibilities across Member State [57]. In this context, shifting collection to hospitals may improve traceability and financing but does not diminish the importance of communication at the pharmacy counter. The present study indicates that pharmacists remain key sources of advice and could be explicitly integrated as communication hubs guiding patients toward hospital-based return options. Pragmatic measures (standardized counseling messages, printed materials, QR codes, and digital reminders) may support this role, particularly when delivered by trusted professionals [7,38,41,42]. For antibiotics, linking disposal guidance to antimicrobial stewardship and pollution-control narratives aligns with WHO priorities [58].

Embedding pharmaceutical waste topics into national guidelines and Continuing Professional Development programs may help normalize waste-related counseling as an integral component of good pharmacy practice. Within the European context, Romania’s centralized approach aligns with broader efforts to clarify institutional responsibilities; however, sustained pharmacist engagement in patient education, adherence support and collaborative prescribing remains essential. Evidence from Sweden, Italy and Germany illustrates the expanding role of pharmacists in sustainability initiatives across both community and hospital settings [59,60,61], while qualitative studies from Scandinavian countries emphasize the importance of clear national guidance and locally adaptable tools to translate environmental awareness into routine professional action [62].

A key strength of this study lies in its timing, capturing community pharmacists’ practices during the early implementation phase of Romania’s revised pharmaceutical waste regulations. The use of a KAP-based analytical structure enabled an integrated assessment of knowledge, attitudes and practices, while the reporting of effect sizes and non-parametric correlations enhanced interpretability beyond reliance on statistical significance alone.

Nevertheless, the cross-sectional and self-reported nature of the data, combined with limited item variability, constrains the explanatory depth of the observed associations and introduces inherent design limitations, which preclude any inference of causality and capture behaviors and attitudes at a single point in time. Findings are based on self-reported responses and may therefore be influenced by recall and social desirability bias. Although no direct identifiers were collected and results were analyzed in aggregate, indirect identification may be theoretically possible in small samples when demographic characteristics are combined. The venue-based convenience sample may introduce selection bias and limits generalizability. Because students could attend the event and the questionnaire did not include a dedicated student category, eligibility was operationalized using self-reported role/qualification and county of practice, and some misclassification cannot be fully excluded. Legislative awareness was also self-reported and not independently verified; therefore, affirmative-response bias may have led to an overestimation of true knowledge of the relevant law and its requirements. Although the survey captured a substantial number of practicing pharmacists within the target administrative area, the results should be interpreted as context-specific and should not be extrapolated nationally or internationally without caution. Differences in local implementation, pharmacy infrastructure, and patient engagement may contribute to variation in counseling opportunities and practices across settings. Finally, the analysis relied on pharmacists’ perspectives only. Future research using broader sampling across multiple regions, longitudinal designs, and complementary data sources (e.g., patient perspectives or objective indicators of counseling and disposal behaviors) would strengthen understanding of how counseling practices evolve as the revised framework matures. Future surveys should also include knowledge-check items to validate self-reported legislative awareness.

Within these limitations, the study provides context-specific evidence that disposal-related counseling practices are shaped not only by knowledge and attitudes but also by perceived demand and system-level changes. As the revised take-back framework continues to develop, pharmacies may retain an important communication role in guiding patients on the safe disposal of unused medicines even in a hospital-centered system. Supporting this role may assist in aligning implementation processes with patient expectations. Further research using broader sampling and longitudinal or objective measures would help assess how counseling practices evolve as the revised framework matures.

## 5. Conclusions

This study provides context-specific evidence from Romania, showing that community pharmacists remain involved in pharmaceutical waste prevention after medicine take-back responsibilities shifted to hospitals under the 2023/2024 framework. Although pharmacies are no longer collection points, pharmacists frequently reported patient interactions on medicine disposal, underscoring the ongoing relevance of the pharmacist–patient interface.

Respondents indicated high awareness of current legislation and positive attitudes toward patient education, but counseling remained largely reactive. Pharmacists who reported more frequent patient questions also provided more proactive guidance, suggesting that patient demand acts as a behavioral cue.

These findings highlight pharmacists’ continuing communication role within the hospital-centered system and the need for standardized guidance and professional training. Broader, longitudinal studies are warranted to examine how counseling practices evolve as implementation progresses.

## Figures and Tables

**Table 1:** Questionnaire items.

Section	Item	Response Format
Section 1: Socio-demographics	1. Age	Open-ended (numeric)
	2. Sex	☐ Male ☐ Female
	3. County of practice	Open-ended (text)
	4. Practice environment	☐ Urban ☐ Rural
	5. Highest educational level completed	☐ Pharmacy assistant ☐ Bachelor ☐ Residency ☐ Master ☐ Doctorate
	6. Professional grade	☐ Pharmacy assistant ☐ Pharmacist ☐ Specialist pharmacist ☐ Primary pharmacist
	7. Years of experience in community pharmacy	☐ <1 year ☐ 1–5 years ☐ 6–10 years ☐ >10 years
	8. Type of pharmacy unit	☐ Drugstore ☐ Independent pharmacy ☐ Local chain ☐ National chain
	9. Position held	☐ Administrator ☐ Head pharmacist ☐ Pharmacist ☐ Pharmacy assistant
Section 2: Practices and roles	10. Do you counsel patients on safe disposal of expired/unused medicines?	☐ Yes ☐ No
	11. Are you approached by patients who wish to return such medicines?	☐ Yes ☐ No
	12. Do you have explicit responsibilities in reducing the amount of expired medicines?	☐ Yes ☐ No ☐ Not sure
	13 *. What types of products expire most frequently in your experience?	☐ Rx ☐ OTC ☐ Supplements ☐ Others: _______
	14 *. What types expire in the largest quantities?	☐ Rx ☐ OTC ☐ Supplements ☐ Others: _______
Section 3: Knowledge and attitudes	15. Are you aware of the current legislation on pharmaceutical waste disposal?	☐ Yes ☐ No
	16. Have you received training on the new regulations?	☐ Yes ☐ No
	17. How often do you provide patients with information on correct disposal?	☐ Never ☐ Rarely ☐ Almost always ☐ Always ☐ Only on request
	18. How often are you asked for such information?	☐ Never ☐ Rarely ☐ Almost always ☐ Always
	19. Do you consider that improper disposal poses health and environmental risks?	☐ Yes ☐ No ☐ Not sure
	20. Do you believe that incorrect disposal of antibiotics contributes to antimicrobial resistance?	☐ Yes ☐ No ☐ Not sure
	21. Is it important to inform patients about correct disposal?	☐ Yes ☐ No ☐ Not sure
	22. Is proactive, in-pharmacy promotion useful?	☐ Yes ☐ No ☐ Not sure

* Items 13–14 were collected but not analyzed/reported in the present manuscript to maintain a narrower focus.

**Table 2:** Socio-demographic and professional characteristics of community pharmacists.

Parameter	Category	n	%
Age (mean ± SD)	—	41.2 ± 9.4	
Sex	Male	25	8.8
	Female	260	91.2
Practice setting	Urban	198	69.5
	Rural	87	30.5
Educational level	Bachelor’s degree	235	82.5
	Master’s degree	23	8.1
	Residency	16	5.6
	Doctorate	11	3.9
Professional grade	Licensed pharmacist	264	92.6
	Specialist pharmacist	10	3.5
	Primary pharmacist	11	3.9
Experience	<1 year	3	1.1
	1–5 years	37	13.0
	6–10 years	12	4.2
	>10 years	233	81.8
Pharmacy type	Independent	75	26.3
	Local chain	121	42.5
	National chain	89	31.2
Position held	Pharmacist	233	81.8
	Head pharmacist	52	18.2

**Table 3:** Knowledge and attitudes regarding pharmaceutical waste management (N = 285).

Item (15–22)	Variable/Response Option	n	%
Awareness of current legislation on pharmaceutical waste disposal	Yes	280	98.2
	No	5	1.8
Received professional training on relevant regulations	Yes	269	94.4
	No	16	5.6
Frequency of providing information to patients on correct disposal	Never	0	0.0
	Rarely	80	28.1
	Almost always	15	5.3
	Always	32	11.2
	Only on request	158	55.4
Frequency of being asked by patients for such information	Never	17	6.0
	Rarely	256	89.8
	Almost always	10	3.5
	Always	2	0.7
Considers improper disposal to pose health and environmental risks	Yes	284	99.6
	Not sure	1	0.4
Believes that incorrect disposal of antibiotics contributes to antimicrobial resistance	Yes	272	95.4
	No	6	2.1
	Not sure	7	2.5
Considers patient information on disposal important	Yes	282	98.9
	Not sure	3	1.1
Considers proactive in-pharmacy promotion useful	Yes	279	97.9
	No	1	0.4
	Not sure	5	1.8

**Table 4:** Distribution of patient request frequency (Q18) by counseling proactivity (Q17) (N = 285).

Patient Asks Frequency (Q18)	Proactive n (%)	Reactive n (%)	Total
Never	7 (5.5)	10 (6.3)	17 (6.0)
Rarely	110 (86.6)	146 (92.4)	256 (89.8)
Almost always	8 (6.3)	2 (1.3)	10 (3.5)
Always	2 (1.6)	0 (0.0)	2 (0.7)
Total	127 (100)	158 (100)	285 (100)

Note: χ^2^(3) = 7.914, *p* = 0.048.

**Table 5:** Proactive vs. reactive counseling by patient request frequency (dichotomized analysis).

Patient Request Frequency	Proactive n (%)	Reactive n (%)	Total	Statistics
Low (Never/Rarely)	117 (42.9)	156 (57.1)	273	χ^2^ (1) = 7.623, *p* = 0.006; Fisher’s exact *p* = 0.007 PR = 1.94 (95% CI 1.46–2.59); RD = +40.5 pp (95% CI +18.6 to +62.4).
High (Almost always/Always)	10 (83.3)	2 (16.7)	12	

PR = prevalence ratio; RD = risk difference; CI = confidence interval.

**Table 6:** Association between declared responsibilities, counseling, and patient requests (N = 280).

Comparison	Group 1 (n = 265) % Yes	Group 2 (n = 15) % Yes	Chi^2^ (*p*)	Cramer’s V
Responsibilities—counseling	98.9% (262/265)	93.3% (14/15)	3.09 (*p* = 0.079)	0.105
Responsibilities—being approached	95.5% (253/265)	93.3% (14/15)	0.15 (*p* = 0.702)	0.023

Group 1—respondents reporting waste-related responsibilities; Group 2—respondents without such responsibilities. Effect size measured by Cramer’s V.

**Table 7:** Spearman’s rank correlations between knowledge, attitudes and practice (N = 285).

Associated Variables	ρ (rho)	*p*
Training received—awareness of legislation	0.548	<0.001
Importance of informing patients—usefulness of proactive promotion	0.706	<0.001
Perceived AMR risk—importance of informing patients	0.321	<0.001
Perceived AMR risk—usefulness of proactive promotion	0.227	<0.001
Frequency of providing information—importance of informing patients	−0.143	0.016

ρ = Spearman’s rank correlation coefficient. All tests two-tailed. *p* < 0.05 considered statistically significant.

## Data Availability

The original contributions presented in this study are included in the article/Supplementary Materials. Further inquiries can be directed to the corresponding authors.

## Supplementary Materials

The following supporting information can be downloaded at: https://www.mdpi.com/article/10.3390/healthcare14030386/s1, Supplementary Material S1: Full Questionnaire.

## Abbreviations

AMR	Antimicrobial resistance
ANMDMR	National Agency for Medicines and Medical Devices of Romania (in Romanian: Agenția Națională a Medicamentului și a Dispozitivelor Medicale din România)
APIs	Active pharmaceutical ingredients
ARB	Antibiotic-resistant bacteria
ARGs	Antibiotic resistance genes
CDC	U.S. Centers for Disease Control and Prevention (referenced for Epi Info™ 7)
CNAS	National Health Insurance House (in Romanian: Casa Națională de Asigurări de Sănătate), Romania
CPD	Continuing Professional Development
FE	Frequently Expiring (products)
HVE	Higher Volume Expiring (products)
KAP	Knowledge, Attitudes, and Practices (surveys/studies)
MS	Ministry of Health (in Romanian: Ministerul Sănătății), Romania
OECD	Organization for Economic Co-operation and Development
OR	Odds Ratio
OTC	Over the Counter (medicines)
QR	Quick Response (code), used for signposting/links to collection points
PR	Prevalence ratios
Rx	Prescription medicines
SD	Standard Deviation
