# Supplementary material for: From Policy to Practice: Community Pharmacists’ Self-Reported Counseling Role in Pharmaceutical Waste Management
Source: Healthcare (Basel). 2026 Feb 3;14(3):386. doi: 10.3390/healthcare14030386 (PMC12897107; doi:10.3390/healthcare14030386)
Supplement: Supplementary file 1 [file healthcare-14-00386-s001.zip › Supplementary Material S1 -Questi onnaire items (Sections 1–3).pdf]

Supplementary Material S1 – Full Questionnaire

Pharmacists' involvement in reducing pharmaceutical waste in community settings
Introductory Statement
This questionnaire is part of an observational study that aims to assess the importance of the pharmacist's and pharmacy assistant's involvement in reducing the amount of pharmaceutical waste (expired or unused medicines resulting from outpatient treatment).
The questionnaire is strictly anonymous; responses are confidential and will never be associated with your identity. The results of this survey will be used solely for research purposes. By completing this questionnaire, I consent to participate in the study and agree to the anonymous use of the data I provide for research purposes.

Initials: _______ Date: ___________

Section 1: General Information

1. Age: ....

2. Sex:

☐ Male ☐ Female

3. County of practice: ....

4. Practice environment:

☐ Urban ☐ Rural

5. Highest educational level completed:

☐ Pharmacy assistant ☐ Bachelor's degree ☐ Residency ☐ Master's degree ☐ Doctorate

6. Professional grade:

☐ Pharmacy assistant ☐ Pharmacist ☐ Specialist pharmacist ☐ Primary pharmacist

7. Years of experience in community pharmacy:

☐ <1 year ☐ 1–5 years ☐ 6–10 years ☐ >10 years

8. Type of pharmacy unit:

☐ Drugstore ☐ Independent pharmacy ☐ Local chain ☐ National chain

9. Position held:

☐ Administrator ☐ Head pharmacist ☐ Pharmacist ☐ Pharmacy assistant

Section 2: Current Practices and Perceived Responsibilities

10. Do you counsel patients on the safe disposal of expired or unused medicines?

☐ Yes ☐ No

11. Are you approached by patients who wish to return such medicines?

☐ Yes ☐ No

1**2. Do you have explicit responsibilities in reducing the amount of expired medicines?**

☐ Yes ☐ No ☐ Not sure

13. What types of products expire most frequently in your experience?

☐ Rx ☐ OTC ☐ Supplements ☐ Other: ....

14. What types expire in the largest quantities?

☐ Rx ☐ OTC ☐ Supplements ☐ Other: ....

Section 3: Knowledge and Attitudes

15. Are you aware of the current legislation on pharmaceutical-waste disposal?

☐ Yes ☐ No

16. Have you received training on the new regulations?

☐ Yes ☐ No

17. How often do you provide patients with information on correct disposal?

☐ Never ☐ Rarely ☐ Almost always ☐ Always ☐ Only on request

18. How often are you asked for such information?

☐ Never ☐ Rarely ☐ Almost always ☐ Always

19. Do you consider that improper disposal poses health and environmental risks?

☐ Yes ☐ No ☐ Not sure

20. Do you believe that incorrect disposal of antibiotics contributes to antimicrobial resistance?

☐ Yes ☐ No ☐ Not sure

21. Is it important to inform patients about correct disposal?

☐ Yes ☐ No ☐ Not sure

22. Is proactive, in-pharmacy promotion useful?

☐ Yes ☐ No ☐ Not sure

Notes:

OTC = Over-the-counter medicines; Rx = Prescription medicines.

No skip logic was applied; all items were presented in the same fixed order to all participants.

The questionnaire was administered in Romanian; this is the English translation for transparency.
